# Occurrence and Dietary Exposure Assessment of Quinolone Antibiotics in Animal-Derived Foods and Associated Health Risks Among Different Population Groups in Guangzhou, China

**DOI:** 10.3390/foods15050848

**Published:** 2026-03-03

**Authors:** Zexian Xie, Yanyan Wang, Yonglin Chen, Yan Li, Yuhua Zhang, Lan Liu, Rongfei Peng, Weiwei Zhang, Yu-Heng Mao

**Affiliations:** 1 School of Exercise and Health, Guangzhou Sport University, Guangzhou 510500, China; 18575697886@163.com (Z.X.); chenyl946@163.com (Y.C.); 2 Guangzhou Center for Disease Control and Prevention, Guangzhou Health Supervision Institute, Guangzhou 510440, China; wangyy13213845@163.com (Y.W.); gzcdcliy@foxmail.com (Y.L.); pisceszyh@126.com (Y.Z.); liulan728@163.com (L.L.); gzprf@126.com (R.P.); 3 Guangdong Provincial Key Laboratory of Human Sports Performance Science, Guangzhou Sport University, Guangzhou 510500, China

**Keywords:** quinolone antibiotics, veterinary drug residues, dietary exposure, health risk assessment, food safety

## Abstract

Quinolone antibiotics (QNs) are widely used in animal production and may pose potential health risks through dietary exposure. A total of 1612 animal-derived food samples covering 10 food categories were collected in Guangzhou, China, from 2016 to 2023. Residues of six QNs were determined using ultra-performance liquid chromatography coupled with tandem mass spectrometry. Dietary exposure among different age groups was assessed using a probabilistic approach based on local food consumption data, and non-carcinogenic health risks were characterized using hazard quotient (HQ) and hazard index (HI) methods. QN residues were detected in 7.75% of samples, with an exceedance rate of 2.23%. Aquatic products, particularly fish and crustaceans, exhibited the highest detection frequencies and contributed most to overall dietary exposure. Enrofloxacin (ENR) was the most frequently detected compound, while sporadic samples showed extremely high residue concentrations (1003 unit/g in eggs). Children aged 3–6 years had the highest HI (mean is 1.94 × 10^−2^). All HQ and HI values were below 1, indicating low non-carcinogenic health risks under current exposure scenarios. Although dietary exposure to QNs among Guangzhou residents is unlikely to pose appreciable non-carcinogenic health risks, elevated exposure in children and sporadic high-residue events highlight the need for continued risk-based monitoring and targeted food safety management.

## 1. Introduction

Antibiotic contamination has evolved into a global environmental and food safety challenge, transcending geographical boundaries, as evidenced by its detection in more than 65% of river sampling sites across 72 countries worldwide [1]. As emerging pollutants, antibiotics have been widely detected in agricultural soils, aquatic environments, and food products, raising significant concerns regarding their ecological impacts and potential risks to human health [2,3]. Among these contaminants, quinolone antibiotics (QNs) are of particular concern due to their extensive use in both human medicine and animal husbandry. QNs exert bactericidal effects primarily by inhibiting bacterial DNA cyclase [4]. Their widespread application, combined with pharmacokinetic properties such as relatively long elimination half-lives (e.g., 6–12 h for commonly used QNs), contributes to their environmental persistence and continuous entry into the food chain [5]. Consequently, residues of QNs in animal-derived foods represent a direct and important route of chronic dietary exposure for the general population.

The extensive and, in some cases, inappropriate use of QNs has accelerated the emergence and dissemination of antimicrobial resistance (AMR), which is now recognized as a major global public health threat. Persistent low-level exposure to antibiotics can exert selective pressure on microbial communities, facilitating the development of resistant bacteria and the horizontal transfer of resistance genes to human microbiota [6,7]. Such processes complicate the treatment of bacterial infections and increase the burden on healthcare systems. Although global antibiotic consumption temporarily declined during the COVID-19 pandemic, a subsequent rebound has been reported, particularly in middle-income countries, underscoring the continued need for effective monitoring, exposure assessment, and risk management strategies [8].

The report indicates that China’s annual antibiotic consumption ranges between 162,000 and 180,000 metric tons, primarily used in human healthcare, animal husbandry, and agriculture [9]. However, compared with regulatory frameworks such as the EU Directive 2021/808, China’s national standard GB 31650-2019 and GB 31650.1-2022 remain relatively limited in terms of detailed analytical requirements, quality assurance measures, and quality control provisions for residue testing [10,11]. These regulatory gaps pose practical challenges for effective oversight in developing regions with high agricultural intensity. The Pearl River Delta, one of the most urbanized and densely populated regions in South China, poses a high risk due to substantial antibiotic demand and intensive food production. Moreover, the region’s hot and humid climate may influence the degradation dynamics and persistence of antibiotic residues in water and soil, thereby increasing the likelihood of environmental contamination and subsequent transfer to food products [12]. As the capital of Guangdong Province and a core city of the Guangdong-Hong Kong-Macao Greater Bay Area, Guangzhou plays a pivotal role in regional food supply, consumption, and food safety governance [13]. In response, local authorities have implemented policy initiatives, such as the Guangzhou Food Safety Supervision and Management Plan 2025, which emphasizes strict control of excessive veterinary drug residues and the illegal use of banned or restricted substances in food [14].

Despite these regulatory efforts, current dietary exposure and risk assessments for antibiotic residues in China remain inadequate. Many assessments continue to rely on outdated dietary consumption data derived from surveys conducted in 2002, which no longer reflect contemporary dietary patterns, food consumption behaviors, or changes in food supply systems [15]. In addition, refined exposure assessment models that account for population heterogeneity are rarely applied. The Joint FAO/WHO Expert Committee on Food Additives (JECFA) has recently emphasized that infants under three years of age and fetuses eliminate exogenous substances at only 1/2–1/3 the rate of adults. However, domestic risk assessments in China for contaminants of emerging concern, including antibiotics, often continue to adopt adult exposure parameters without incorporating updated weighting factors or establishing specific exposure scenarios for vulnerable populations such as children and pregnant women. This practice may lead to a systematic underestimation of dietary exposure and associated health risks [16].

Therefore, the present study aims to systematically quantify QN residues in major animal-derived foods consumed daily by residents across the 11 administrative districts of Guangzhou. By integrating updated food consumption data with refined dietary exposure assessment models, this study seeks to (1) characterize the levels and distribution patterns of QN residues in commonly consumed foods of animal origin, (2) identify high-risk food categories, vulnerable sub-populations, and critical geographical distribution points, and (3) provide a scientific basis for targeted risk management strategies and collaborative food safety governance within the Guangdong-Hong Kong-Marcao Greater Bay Area.

## 2. Materials and Methods

### 2.1. Food Sampling

All samples were collected between May 2016 and October 2023 from six types of sales outlets (including eateries, farms, farmers’ markets, fresh markets, living supermarkets, and online stores) across 11 administrative districts of Guangzhou, Guangdong Province, China (Table A1). Eateries refer to establishments that provide food and beverage services. A farm is a place where livestock are raised. A farmers’ market is a market established in urban or rural areas where agricultural products and byproducts can be bought and sold freely. Fresh markets are supermarkets specializing in fresh food products such as vegetables, fruits, meat, seafood, poultry, and eggs. A living supermarket is a large retail store that operates on a self-service model, primarily selling food, daily necessities, and other goods. An online store is a commercial establishment established on an online platform using digital information technology. A stratified random sampling strategy was adopted based on street-level information from local government authorities. In each district, three streets (two central urban streets and one remote/suburban street) were selected using computer-generated random numbers. In total, 33 streets (22 central and 11 remote) were selected as sampling locations. This design prioritizes the relevance of exposure over artificial uniformity. Common farmers’ markets and fresh produce markets naturally offer more samples. Food samples were purchased from multiple sales points by trained investigators following standardized sampling procedures. Additionally, sample sizes for different food categories may vary due to consumer-driven purchasing patterns. For instance, eggs and chicken are more commonly consumed by residents in Guangzhou, resulting in correspondingly larger sample sizes for these food categories. Since the compounds under analysis (QNs) are all qualitative compounds expected to be present only in animal-derived foods, sampling was primarily conducted on animal-derived foods. Overall, 1612 food samples representing different animal-derived food categories (including beef, chicken, crustaceans, duck, eggs, fish, milk, mollusks, pork, and viscera) were collected and transported to the laboratory under refrigerated conditions for further analysis.

### 2.2. Sample Preparation

Animal muscle and livestock offal samples were thawed at room temperature, trimmed of visible connective tissue, and homogenized thoroughly. Milk samples were thawed and mixed uniformly. Egg samples were shelled and homogenized prior to analysis. All prepared samples were stored at −20 °C until extraction.

### 2.3. Extraction and Purification

QN residues were extracted following a modified acetonitrile-based extraction procedure. Briefly, 2.0 g (±0.001 g) of the homogenized samples was weighed into a 50 mL centrifuge tube, and 10 mL of acidified acetonitrile-water solution (*v*/*v*/*v*: 1/84/15) was added for extraction. The mixture was vortex-mixed for 3 min, sonicated for 10 min, and centrifuged at 9000 r/min for 5 min.

For purification, 5.0 mL of the supernatant was transferred into a 15 mL centrifuge tube containing 250 mg DSC-C18 sorbent. The mixture was vortexed for 1 min, sonicated for 10 min, and centrifuged at 9000 r/min for 5 min. Subsequently, 1.0 mL of the purified supernatant was diluted with 3.0 mL of 0.1% formic acid aqueous solution and filtered through a 0.22 μm microporous membrane prior to ultra-performance liquid chromatography coupled with tandem mass spectrometry (UPLC–MS/MS).

### 2.4. UPLC-MS/MS Analysis

Quantification of QN residues was performed using UPLC-MS/MS. Chromatographic separation was achieved on a C18 column (Waters AcQuity TQ-S Micro, AB SCIEX, Marlborough, MA, USA, 150 × 3 mm, 3 μm particle size). The mobile phase consisted of solvent A (acetonitrile containing 0.15% formic acid) and solvent B (water containing 0.15% formic acid). The gradient elution program is summarized in Table 1. The flow rate was set at 0.50 mL/min, with a total run time of 11 min. The injection volume was 1.0 µL, and the column temperature was maintained at 30 °C.

Mass spectrometric detection was conducted in positive electrospray ionization (ESI+) mode with the following parameters: capillary voltage, 2.0 kV; cone voltage, 27 V; source temperature, 150 °C; desolvation temperature, 500 °C; desolvation gas flow rate, 800 L/h; and cone gas flow rate, 50 L/h. Multiple reaction monitoring transitions and optimized MS parameters for individual QNs are listed in Table 2.

### 2.5. Calibration Curves

Mixed standard stock solutions of QNs (10 μg/mL) were prepared in acetonitrile. A working standard solution (1 μg/mL) was prepared by appropriate dilution and further diluted to 100 μg/L and 10 μg/L.

Aliquots of the standard solution were diluted with the initial mobile phase to prepare calibration standards at six concentration levels (0.3, 1.0, 5.0, 10, 25, and 50 μg/L). Reagent blanks were prepared simultaneously.

Calibration curves were constructed by plotting peak areas against corresponding concentrations. All QNs exhibited good linearity over the tested range, with correlation coefficients (r) between 0.999103 and 0.999917.

### 2.6. Quality Control and Method Validation

The limit of detection (LOD) and limit of quantification (LOQ) were defined at signal-to-noise ratios (S/N) of 3:10, respectively. Method accuracy and precision were evaluated by spiking QN-free animal tissue samples at six concentration levels (0.3–50 μg/L).

Recoveries for enrofloxacin (ENR), ciprofloxacin (CIP), ofloxacin (OFL), prefloxacin (PFLX), norfloxacin (NOR), and lomefloxacin (LOM)) ranged from 84.4–104%, 87.7–107%, 93.8–109%, 95.2–108%, 79.0–102%, and 95.0–99.5%, respectively. The LOD and LOQ are adjusted annually in accordance with national government requirements. The ranges for the LOD and LOQ of the six QNs are 0.10–3.00 μg/kg and 0.33–10.00 μg/kg, respectively.

### 2.7. Dietary Consumption Data

Dietary consumption data were obtained from a Guangzhou-wide food consumption survey conducted in 2011, covering both urban and suburban populations. Food intake was assessed using three consecutive 24-h dietary recall questionnaires. Detailed survey procedures have been described previously [17].

In brief, 2960 residents (1416 males and 1544 females) from 998 households participated in the survey, including 1888 urban residents (63.8%) and 1072 suburban residents (36.2%). Participants were categorized into four age groups: 3–6 years, 7–17 years, 18–59 years, and ≥60 years, accounting for 6.7%, 21.5%, 58.6%, and 13.2% of the population, respectively [18,19,20,21].

### 2.8. Dietary Exposure and Risk Assessment

Dietary exposure to QNs among Guangzhou residents was assessed using a chronic dietary exposure framework. The assessment focused on non-carcinogenic health effects associated with long-term intake of QNs through the consumption of animal-derived foods. Estimated daily intake (EDI) was calculated for different population groups based on measured residue concentrations in foods, age-specific food consumption data, and body weight.

The EDI of individual QNs was calculated according to Equation (1):

(1) $$\mathrm{EDI}=\sum_{i=1}^{n}M_{i}\times D_{i}/M$$

where EDI (μg/kg·bw) represents the estimated daily intake of a specific QN; *M_i_* is the mean concentration of QNs in food category *i* (μg/kg), *D_i_* is the daily consumption of food category *i* (g/d), and *W* is the average body weight of the population group (kg). The food consumption data were derived from the Guangzhou food consumption survey conducted in 2011, which represents the most recent systematic dietary survey available for the study area and is commonly used for long-term dietary exposure assessment. Due to the continuous rise in global obesity rates over the past decade, we have adopted the latest weight reference values. Average body weights were assumed to be 20 kg (3–6 years) [22], 40 kg (7–17 years) [23], 62 kg (18–59 years) [17], and 60 kg (≥60 years and the total) [24,25].

To better characterize variability and uncertainty in dietary exposure, a probabilistic approach was adopted. Monte Carlo simulations were performed using @RISK software (Palisade Corporation, 7.6. Industrial, 2018, Ithaca, NY, USA), integrating distributions of food consumption rates and residue concentrations. For concentrations below the LOD, values were substituted with LOD/2 [21], a commonly applied approach in dietary exposure assessments that aims to minimize potential bias associated with non-detects. A total of 10,000 iterations was conducted for each population group to generate stable exposure distributions.

The non-carcinogenic health risks associated with dietary exposure to individual QNs were characterized using the hazard quotient (HQ), calculated as the ratio of EDI to the acceptable daily intake (ADI), according to Equation (2):

(2) HQ = EDI/ADI

HQ < l indicates that the estimated exposure is unlikely to pose an appreciable non-carcinogenic health risk, whereas HQ > 1 suggests a potential health concern. ADI values are present in Table 3. The European Medicines Agency (EMA) has determined the enrofloxacin ADI as 6.2 μg/kg [26]. However, the EMA has not specified ADIs for the other QNs examined in this study. Furthermore, as our research aims to inform food safety risk management within the Chinese regulatory framework, we primarily reference the Chinese national standards (GB 31650-2019 and GB 31650.1-2022 [10,11]), which are the legally binding references for veterinary drug residues in China.

To evaluate the combined health risk from exposure to multiple QNs, the hazard index (HI) was calculated as the sum of HQs for all QNs, assuming cumulative exposure risk. The HI was calculated according to Equation (3):

(3) $$\mathrm{HI}=\sum_{i=1}^{n}{\mathrm{HQ}}_{n}^{i}$$

HI > 1 indicates that exposure to total QNs poses a health risk.

## 3. Results and Discussion

### 3.1. Overall Occurrence of QN Residues in Foods

A total of 1612 food samples across 10 food categories were collected from supermarkets, fresh markets, farmers’ markets, and other retail outlets in Guangzhou. The overall occurrence of QN residues across different food categories is summarized in Table 4 and Table A2, which highlights substantial differences in detection and exceedance rates among food types. A sample was considered positive if the residue concentration was ≥LOD. Overall, 125 samples were positive for at least one QN, corresponding to an overall detection rate of 7.75%, while 36 samples exceeded the established maximum residue limits (MRLs), resulting in an exceedance rate of 2.23%.

QN residues were detected in 8 out of 10 food categories analyzed. Aquatic products exhibited notably higher detection frequencies, with fish showing the highest detection rate (35.00%), followed by crustaceans (26.67%) and viscera (14.81%). While the highest exceedance rates were most frequently observed in crustaceans (6.67%), eggs (3.86%), and fish (3.33%), indicating that aquatic products and eggs constitute an important source of dietary exposure to QNs. The relatively high detection frequency observed in aquatic products may be associated with the extensive use of QNs in aquaculture and their persistence in aquatic environments [28].

Eggs and chicken also showed notable detection and exceedance rates. Eggs presented a detection rate of 4.03% and an exceedance rate of 3.86%, primarily associated with ENR, CIP, and OFL. Chicken samples showed a detection rate of 6.76% and an exceedance rate of 1.47%, mainly due to OFL and PFLX residues.

In contrast, no QN residues were detected in gastropods, milk products, beef, meat products, and amphibian reptiles, suggesting relatively effective control of QNs in these food categories during the monitoring period.

### 3.2. Occurrence of Individual QN Residues in Different Food Categories

**Figure 1:**
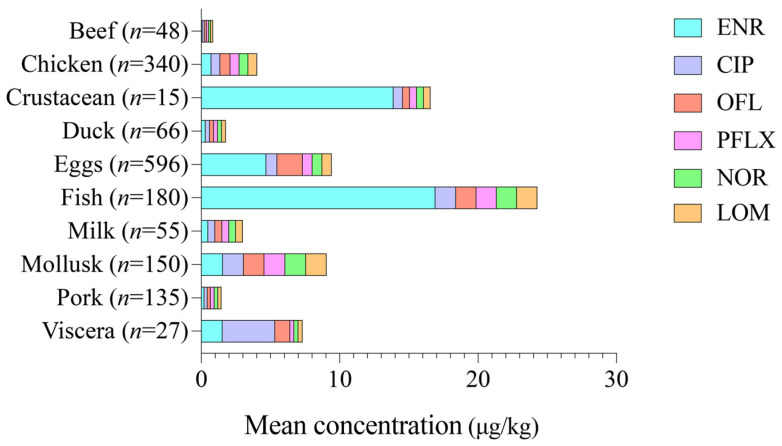
The occurrence and distribution of QNs in different food categories. ENR: enrofloxacin; CIP: ciprofloxacin; OFL: ofloxacin; PFLX: pefloxacin; NOR: norfloxacin; LOM: lomefloxacin.

The occurrence of individual QNs varied markedly across food categories as summarized in Table 4, and their concentration distributions are further illustrated in Figure 1. Among the six QNs investigated, LOM was not detected in any of the analyzed samples, whereas the remaining five QNs were detected in at least one food category. ENR was the most frequently detected compound, with an overall detection rate of 6.76%, followed by OFL (0.93%) and CIP (0.56%). PFLX and NOR were detected less frequently. PFLX was detected in only one chicken sample. NOR was detected in only one pork sample.

ENR was predominantly detected in fish, chicken, and eggs, accounting for the majority of positive samples in these categories. CIP was mainly detected in viscera, eggs, fish, and duck samples, while OFL was primarily observed in eggs and chicken, with overall detection rates of 0.56% for CIP and 0.93% for OFL. PFLX was detected only in chicken samples, and NOR was detected exclusively in pork samples.

As shown in Figure 1, fish exhibited the highest ENR residue levels, followed by crustaceans and eggs. Viscera showed relatively high CIP residue levels compared with other food categories, while elevated OFL residues were mainly observed in eggs and fish.

In fish samples, the mean ENR concentration was 16.92 μg/kg, with a maximum concentration of 301.00 μg/kg. Notably, four freshwater fish samples contained ENR concentrations exceeding 200 μg/kg, surpassing the MRLs by more than two-fold. In crustaceans, the mean ENR concentration was 13.89 μg/kg, with a maximum concentration of 97.80 μg/kg, approaching the regulatory limit.

Egg samples showed substantial variability in residue levels. The mean ENR concentration was 4.69 μg/kg, with a maximum of 1003.00 μg/kg. Two egg samples contained ENR concentrations of 1003.00 μg/kg and 770.00 μg/kg, accompanied by CIP concentrations of 30.00 μg/kg and 12.50 μg/kg, respectively, exceeding the combined MRLs for ENR and CIP by more than two orders of magnitude. In addition, one egg sample contained OFL at 650.00 μg/kg, which was 325 times the corresponding MRL.

In chicken samples, the mean ENR and OFL concentrations were 0.72 μg/kg and 0.74 μg/kg, respectively. One chicken sample contained PFLX at 7.30 μg/kg, exceeding the MRL of 2 μg/kg. Although the average residue levels of QNs in most food categories were low, the presence of extremely high concentrations in a small number of samples suggests improper or illegal use of QNs at the production stage.

### 3.3. Occurrence of QN Residues in Foods with Different Packaging Types

In total, 1293 bulk food samples and 319 pre-packaged food samples were collected in this study. The detection rates and exceedance rates of QN residues in foods with different packaging types are summarized in Table 5. Certain food categories, including crustaceans, duck, mollusks, and viscera, were sampled exclusively in bulk form and were therefore excluded from statistical comparisons between packaging types.

Statistical comparisons were performed for food categories available in both bulk and pre-packaged forms. A significantly higher detection rate and exceedance rate were observed in bulk eggs compared with pre-packaged eggs (*p* = 0.021 and *p* = 0.028, respectively). In contrast, no statistically significant differences were identified between bulk and pre-packaged products for chicken, fish, or pork (*p* > 0.05).

At the aggregate level, the overall detection rate of QNs in bulk foods (9.13%) was significantly higher than that in pre-packaged foods (2.19%) (*p* < 0.001). However, no significant difference in exceedance rates was observed between bulk foods (2.47%) and pre-packaged foods (1.25%) (*p* = 0.186). This higher detection frequency in bulk foods suggests a greater likelihood of QN residues, which may be related to stricter regulatory requirements for prepackaged foods [29].

It should be noted that the number of pre-packaged samples was relatively limited for certain food categories, which may have reduced the statistical power of the comparisons. Nevertheless, the observed differences indicate that packaging type may influence the occurrence of QN residues and should be considered in food safety surveillance programs. These findings suggest that bulk foods may be associated with a higher likelihood of QN occurrence, highlighting the need for enhanced surveillance and traceability measures in bulk food supply chains.

### 3.4. Occurrence of QN Residues in Foods from Different Sampling Sites

The occurrence of QN residues in foods collected from six sampling sites was analyzed, and the detection rates of individual QNs are presented in Table 6. No QN residues were detected in samples collected from eateries or farms, which may be partially attributable to the limited sample size and stricter on-site management practices.

Among six sampling sites, farmers’ markets showed the greatest diversity of detected QNs, with five compounds identified: ENR, CIP, OFL, PFLX, and NOR. Fresh markets had the highest ENR detection rate (16.84%), while supermarkets had detectable levels of both ENR and OFL.

**Figure 2:**
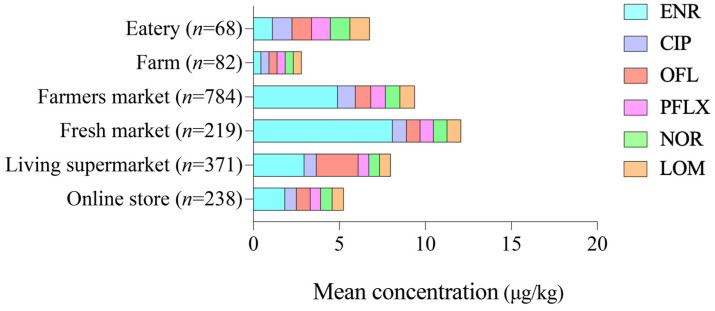
The occurrence and distribution of QNs across different sampling sites. ENR: enrofloxacin; CIP: ciprofloxacin; OFL: ofloxacin; PFLX: pefloxacin; NOR: norfloxacin; LOM: lomefloxacin.

As illustrated in Figure 2, food samples from farmers’ and fresh markets exhibited not only higher detection frequencies of ENR, but also higher average residue concentrations compared with other sampling sites. In contrast, foods collected from farms showed the lowest contamination levels.

In farmers’ markets. ENR, CIP, and OFL were detected at rates of 7.01%, 0.84%, and 1.26%, respectively, with maximum concentrations reaching 1003.00 μg/kg for ENR, 88.20 μg/kg for CIP, and 21.60 μg/kg for OFL (Table A3). Fresh markets showed the highest average ENR concentration (8.10 μg/kg), while the other QNs had average concentrations around 0.65. In living supermarkets, OFL exhibited the highest average concentration among the detected QNs.

These findings indicate that farmers’ markets and fresh markets should be prioritized in future monitoring programs, particularly for ENR contamination, to further reduce dietary exposure risks associated with QN residues.

### 3.5. Comparison with Previous Studies

Several studies have reported the occurrence of QN residues in aquatic products from different regions of China in recent years. For example, Wang et al. [30] investigated QN residues in cultured fish collected from Shandong Province in 2021. Their results showed that QNs were detected in 43.1% of 160 cultured fish samples, with ENR exhibiting both the highest detection frequency and the highest residue concentrations. Similarly, a survey conducted in Fujian Province between 2020 and 2022 reported a QN detection rate of 35.77% in fish samples, with maximum residue levels of 246 μg/kg for ENR and 6.09 μg/kg for OFL [31].

In comparison, the detection rate of QNs in fish samples collected in Guangzhou in the present study was 35.00% (Table 4), which was slightly lower than that reported in Shandong, but comparable to the level observed in Fujian. However, the maximum ENR concentration detected in Guangzhou fish (301.00 μg/kg) exceeded the corresponding values reported in both studies, indicating sporadic but severe contamination events.

In 2019, Fei et al. [32] investigated QN residues in 1754 chicken samples and 1712 pork samples collected from 25 provinces across China. The study reported detection frequencies of 3.45% for chicken and 4.05% for pork samples from Guangdong Province. In contrast, the detection rate of QNs in pork observed in the present study (1.48%; Table 4) was notably lower, suggesting that QN residue risks in pork may vary substantially among different cities within Guangdong Province. Conversely, the detection rate of QNs in chicken samples from Guangzhou (6.76%) was higher than that reported by Wang et al. [30] for Guangdong Province overall, which may be attributable to differences in sampling periods, production systems, or market structures.

Internationally, markedly higher detection rates of QNs have been reported in some regions. In the Kathmandu Valley, Nepal, QNs were detected in 88.33% of chicken samples and 80% of egg samples, with 9 chicken and 3 egg samples exceeding the MRLs [33]. In Lebanon, QNs were detected in 32.5% of chicken meat samples collected from farms in different regions, with CIP being the most frequently detected compound [34]. A study from Indonesia reported detection rates of 41.8% for ENR and 67.3% for CIP in chicken samples, with maximum concentrations of 242.40 ng/g and 275.00 ng/g, respectively [35].

Compared with these international studies, the overall detection rates of QNs in foods from Guangzhou were substantially lower, suggesting relatively stricter regulation and more effective management of QNs use in animal production. Nevertheless, although the general occurrence of QN residues in foods from Guangzhou was lower than that reported in many national and international studies, the presence of high-concentration residues in individual samples indicates persistent risks associated with improper QN use, highlighting the need for continued comprehensive nationwide surveillance.

### 3.6. Health Risk Assessment

#### 3.6.1. Estimated Daily Intake

In the present study, dietary exposure to QNs was assessed based on measured residue levels in foods consumed by different population groups, including children, adolescents, adults, and the elderly. Because LOM was not detected in all foods, and only one sample contained NOR and one sample contained PFLX, the available data were insufficient to reliably evaluate the dietary health risks associated with these compounds.

Dietary consumption data were obtained from a food survey conducted among 2976 Guangzhou residents in 2011, and the consumption of different food categories by each age group is presented in Table A4. The EDI values of individual QNs across 10 food categories for the four age groups are shown in Table A5. Overall, the average EDI of ENR + CIP was the highest among the analyzed QNs, followed by PFLX, OFL, NOR, and LOM, reflecting both their relatively higher detection frequencies and residue levels in foods.

The average EDI values for most QNs generally showed a decreasing trend with increasing age, with a slight increase observed in individuals aged ≥ 60 years. Notably, children aged 3–6 years consistently exhibited higher EDI levels than adolescents and adults. This age-related pattern was particularly pronounced for chicken, eggs, fish, and pork, which are commonly consumed animal-derived foods. Across all population groups, the average EDIs of the six individual QNs ranged from 3.67 × 10^−4^ to 3.10 × 10^−2^ ng/kg·d for chicken, 3.59 × 10^−4^ to 1.40 × 10^−2^ ng/kg∙d for eggs, and 1.05 × 10^−3^ to 3.30 × 10^−2^ ng/kg∙d for fish.

The total dietary intake of QNs among Guangzhou residents was strongly influenced by both the consumption quantity of fish and the relatively higher residue levels detected in fish, making fish the primary contributor to overall dietary QN exposure. Although pork was consumed in the highest quantities, QN exposure from pork remained low due to generally low residue levels, with mean EDIs ranging from 3.60 × 10^−4^ to 1.37 × 10^−3^ ng/kg∙d for the six QNs. In contrast, despite low QN residue levels in milk, its relatively high consumption (63.4 ± 88.1 g/d) resulted in a non-negligible contribution to total dietary QN exposure, highlighting the importance of considering both residue levels and consumption patterns in dietary exposure assessments.

#### 3.6.2. Risk Characterization

**Figure 3:**
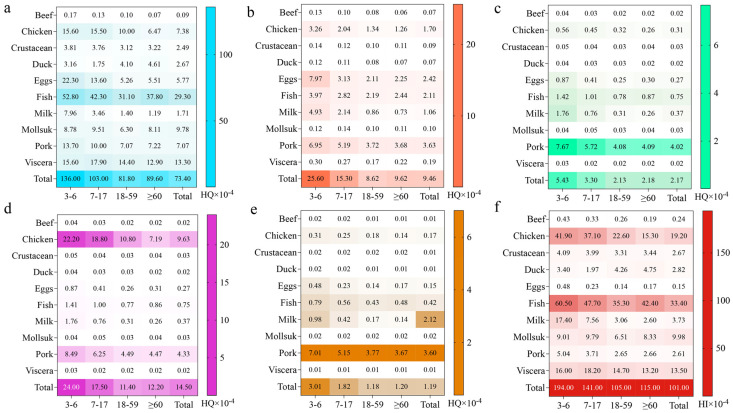
Risk characterization of QN exposure in Guangzhou residents at different ages by HQ analysis. Note: (**a**–**e**) represents the HQs for ENR + CIP, OFL, PFLX, NOR, and LOM, respectively. (**f**) represents HI.

The non-carcinogenic health risks associated with dietary exposure to individual QNs and total QNs were evaluated based on the ADI, and the results are summarized in Table A6, Table A7, and Figure 3. Among these, Figure 3a–e represents the HQs for ENR + CIP, OFL, PFLX, NOR, and LOM, respectively. Figure 3f represents HI. The average HI values were 1.94 × 10^−2^ for the 3–6 age group, 1.41 × 10^−2^ for the 7–17 age group, 1.05 × 10^−2^ for the 18–59 age group, and 1.15 × 10^−2^ for individuals aged ≥ 60 years. The 95th percentile HI values ranged from 3.65 × 10^−2^ to 6.56 × 10^−2^, all of which were well below the threshold value of 1.

Consistent with the EDI results, the average HQ values for QNs generally decreased with increasing age, with the highest values observed in preschool children aged 3 to 6 years. This group exhibited nearly twice the exposure levels compared with other age groups, which can be attributed to their higher food intake relative to body weight. Among the different food categories, fish and chicken contributed relatively higher HQ values than other foods, reflecting both their higher residue concentrations and their importance in the local diet.

Based on these findings, the residues of QNs in animal-derived foods pose no health risk to residents of Guangzhou. Nevertheless, the consistently higher exposure observed in young children and the presence of sporadic high residue concentrations in certain food samples suggest that low-level QN exposure should not be overlooked. Continuous monitoring of QN residues in foods commonly consumed by vulnerable population groups remains important to ensure long-term food safety.

#### 3.6.3. Uncertainty Analysis

Several sources of uncertainty should be acknowledged in the dietary exposure assessment conducted in this study. First, the food categories included did not cover all potential sources of QN contamination; Certain foods that may contribute to QN exposure, such as vegetables and honey [36,37], were not analyzed, which may have led to an underestimation of total dietary exposure. In addition, exposure pathways other than food intake, including water and ambient air, were not considered in the present assessment. Nevertheless, the samples collected in this study are representative and remain highly significant for assessing overall risk.

Second, this study employed a stratified random sampling strategy, primarily based on local food consumption data (to support exposure assessment), while ensuring coverage of various food categories and points of sale to maintain market representativeness for monitoring purposes. Consequently, foods with higher consumption levels (such as chicken, pork, and fish) are sampled more intensively, while foods consumed less frequently (such as crustaceans and viscera) are included with smaller sample sizes to provide basic occurrence information. This compromise may introduce uncertainty regarding the representativeness of monitoring for certain food categories. Additionally, the number of samples collected for each food category was uneven, and for some foods, only one packaging type was available. This limitation may have reduced the statistical power of comparisons between bulk and pre-packaged foods and introduced additional uncertainty into the estimation of occurrence patterns.

Finally, dietary intake data were derived from a 2011 food survey of 2976 Guangzhou residents. This dataset is relatively outdated and may not adequately reflect dietary patterns and consumption behaviors during 2016–2023, particularly considering the rapid urbanization, transformations in the food supply system, and evolving dietary preferences over the past decade. The absence of a recent comprehensive dietary survey in Guangzhou represents a significant data limitation of this study. Future research should prioritize conducting updated dietary assessments to enhance the accuracy and relevance of exposure evaluations.

Despite these limitations, the exposure assessment was based on long-term food consumption data and residue monitoring results collected over multiple years, thereby enhancing the robustness of the overall risk characterization. This study assessed chronic (long-term) dietary exposure to QNs and found no appreciable health risk under current consumption scenarios. However, acute exposure scenarios (e.g., high-consumption events or consumption of highly contaminated single samples) were not evaluated and warrant further investigation. Continuous monitoring and the inclusion of a broader range of food categories in future studies are therefore warranted to further reduce uncertainty and improve the accuracy of dietary risk assessments [33].

## 4. Conclusions

This study provides a comprehensive assessment of the occurrence and dietary exposure risks of QN residues in foods marketed in Guangzhou. The monitoring results indicate that QN residues remain detectable in multiple food categories, with sporadic exceedances of maximum residue limits, suggesting that the improper or non-compliant use of veterinary drugs has not been fully eliminated. Although the overall dietary exposure and associated health risks were evaluated as low for the general population, young children, in particular, exhibited relatively higher exposure levels.

Based on the combined findings of residue monitoring and dietary risk assessment, the results underscore the importance of strengthening regulatory oversight of veterinary drug use in animal production systems. Targeted measures, including improved standardization of drug application practices, enhanced traceability, and timely data sharing of monitoring outcomes, are essential for effective risk management. The development and implementation of alternative disease control strategies, such as non-antibiotic approaches in animal husbandry, may further contribute to reducing reliance on QNs and mitigating residue risks.

Overall, this study provides scientific evidence to support risk-based food safety supervision and highlights the need for continuous monitoring of veterinary drug residues to ensure long-term consumer protection and public health.

## Figures and Tables

**Table 1:** The procedure for gradient elution.

Time (min)	Mobile Phase A	Mobile Phase B
	Acetonitrile + 0.15% Formic Acid	Water + 0.15% Formic Acid
0	80	20
1	80	20
10	100	0
11	100	0

**Table 2:** UPLC-MS/MS parameters for six QNs.

QNs	RT (min)	Precursor Ion (m/z)	Product Ion (m/z)	Collision Energy (eV)	Cone-Hole Voltage (V)
ENR	5.84	360.2	245.0 *	26	40
			316.1	20	40
CIP	5.32	332.1	231.1 *	35	40
			314.1	30	40
OFL	5.04	362.1	261.1 *	26	40
			318.1	20	40
PFLX	5.14	334.1	290.1 *	20	40
			316.1	20	40
NOR	5.08	320.1	233.0 *	25	40
			302.0	19	40
LOM	5.66	352.1	265.1 *	22	35
			308.1	16	35

Note: UPLC-MS/MS: ultra-performance liquid chromatography coupled with tandem mass spectrometry; Band * is the quantitative ion; ENR: enrofloxacin; CIP: ciprofloxacin; OFL: ofloxacin; PFLX: pefloxacin; NOR: norfloxacin; LOM: lomefloxacin.

**Table 3:** ADI and MRLs of various QNs.

QNs	Residual Marker	MRLs (2016–2022 Years)	MRLs (2022 Years–Now)	ADI	Reference
ENR	ENR + CIP	100 μg/kg (eggs and egg products are 0 μg/kg)	100 μg/kg (eggs and egg products are 10 μg/kg)	0–6.2 μg/kg·bw *	[10]
OFL	OFL	0 μg/kg	2 μg/kg	0–5 μg/kg·bw	[11]
NOR	NOR	0 μg/kg	2 μg/kg	0–14 μg/kg·bw	[27]
PFLX	PFLX	0 μg/kg	2 μg/kg	0–14 μg/kg·bw	[11]
LOM	LOM	0 μg/kg	2 μg/kg	0–25 μg/kg·bw	[11]

Note: ADI: acceptable daily intake; MRLs: maximum residue limits; QNs: quinolone antibiotics; *: body weight; ENR: enrofloxacin; CIP: ciprofloxacin; OFL: ofloxacin; PFLX: pefloxacin; NOR: norfloxacin; LOM: lomefloxacin.

**Table 4:** Quinolone residues (total and individual) detected in various foods from Guangzhou, 2016–2023 (unit: μg/kg).

Food Category (*n* = Sampling Number)	Statistic	ENR	CIP	OFL	PFLX	NOR	LOM	At Least One QNs Detected [% (*n*)]	At Least One QNs Exceeded [% (*n*)]
Total (*n* = 1612)	Detection frequency [% (*n*)]	6.76% (109)	0.56% (9)	0.93% (15)	0.06% (1)	0.06% (1)	0% (0)	7.75% (125)	2.23% (36)
	Mean	4.13	0.86	1.22	0.75	0.76	0.76		
	SD	36.12	2.44	16.20	0.59	0.58	0.57		
	Min	0.299	0.8	0.7	<LOD	<LOD	<LOD		
	Max	1003	88.2	650	7.3	3.64	<LOD		
Beef (*n* = 48)	Detection rate [% (*n*)]	0% (0)	0% (0)	0% (0)	0% (0)	0% (0)	0% (0)	0% (0)	0% (0)
	Mean	0.09	0.15	0.15	0.15	0.15	0.15		
	SD	0.05	0.00	0.00	0.00	0.00	0.00		
	Min	<LOD	<LOD	<LOD	<LOD	<LOD	<LOD		
	Max	<LOD	<LOD	<LOD	<LOD	<LOD	<LOD		
Chicken (*n* = 340)	Detection rate [% (*n*)]	5.29% (18)	0% (0)	1.18% (4)	0.29% (1)	0% (0)	0% (0)	6.76% (23)	1.47% (5)
	Mean	0.72	0.64	0.74	0.66	0.64	0.64		
	SD	0.70	0.55	1.65	0.66	0.55	0.55		
	Min	0.30	<LOD	1.50	<LOD	<LOD	<LOD		
	Max	5.60	<LOD	29.40	7.30	<LOD	<LOD		
Crustaceans (*n* = 15)	Detection rate [% (*n*)]	26.67% (4)	6.67% (1)	0% (0)	0% (0)	0% (0)	0% (0)	26.67% (4)	6.67% (1)
	Mean	13.89	0.68	0.50	0.50	0.50	0.50		
	SD	28.20	0.66	0.00	0.00	0.00	0.00		
	Min	4.91	<LOD	<LOD	<LOD	<LOD	<LOD		
	Max	97.80	3.13	<LOD	<LOD	<LOD	<LOD		
Duck (*n* = 66)	Detection rate [% (*n*)]	3.03% (2)	1.51% (1)	0% (0)	0% (0)	0% (0)		3.03% (2)	0% (0)
	Mean	0.32	0.30	0.29	0.29	0.29	0.29		
	SD	0.21	0.11	0.00	0.00	0.09	0.09		
	Min	0.79	<LOD	<LOD	<LOD	<LOD	<LOD		
	Max	1.80	0.80	<LOD	<LOD	<LOD	<LOD		
Eggs (*n* = 596)	Detection rate [% (*n*)]	2.68% (16)	0.50% (3)	16.78% (10)	0% (0)	0% (0)	0% (0)	4.03% (24)	3.86% (23)
	Mean	4.69	0.80	1.84	0.70	0.70	0.70		
	SD	54.00	1.62	26.58	0.50	0.50	0.50		
	Min	0.42	12.50	0.70	<LOD	<LOD	<LOD		
	Max	1003.00	30.00	650.00	<LOD	<LOD	<LOD		
Fish (*n* = 180)	Detection rate [% (*n*)]	35.00% (63)	0.56% (1)	0% (0)	0% (0)	0% (0)	0% (0)	35.00% (63)	3.33% (6)
	Mean	16.92	1.49	1.47	1.46	1.47	1.47		
	SD	41.59	0.26	0.16	0.19	0.16	0.16		
	Min	1.50	4.26	<LOD	<LOD	<LOD	<LOD		
	Max	301.00	4.26	<LOD	<LOD	<LOD	<LOD		
Milk (*n* = 55)	Detection rate [% (*n*)]	0% (0)	0% (0)	0% (0)	0% (0)	0% (0)	0% (0)	0% (0)	0% (0)
	Mean	0.50	0.50	0.50	0.50	0.50	0.50		
	SD	0.00	0.00	0.00	0.00	0.00	0.00		
	Min	<LOD	<LOD	<LOD	<LOD	<LOD	<LOD		
	Max	<LOD	<LOD	<LOD	<LOD	<LOD	<LOD		
Mollusk (*n* = 150)	Detection rate [% (*n*)]	2.00% (3)	0% (0)	0% (0)	0% (0)	0% (0)	0% (0)	2.00% (3)	0% (0)
	Mean	1.56	1.50	1.50	1.50	1.50	1.50		
	SD	0.40	0.00	0.00	0.00	0.00	0.00		
	Min	3.20	<LOD	<LOD	<LOD	<LOD	<LOD		
	Max	5.20	<LOD	<LOD	<LOD	<LOD	<LOD		
Pork (*n* = 135)	Detection rate [% (*n*)]	0.74% (1)	0% (0)	0% (0)	0% (0)	0.74% (1)	0% (0)	1.48% (2)	0.74% (1)
	Mean	0.22	0.23	0.23	0.28	0.26	0.23		
	SD	0.27	0.25	0.25	0.31	0.39	0.25		
	Min	<LOD	<LOD	<LOD	<LOD	<LOD	<LOD		
	Max	0.92	<LOD	<LOD	<LOD	3.64	<LOD		
Viscera (*n* = 27)	Detection rate [% (*n*)]	7.41% (2)	11.11% (3)	3.70% (1)	0% (0)	0% (0)	0% (0)	14.81% (4)	0% (0)
	Mean	1.54	3.79	1.08	0.30	0.30	0.30		
	SD	4.46	16.60	4.03	0.10	0.10	0.10		
	Min	16	1.12	<LOD	<LOD	<LOD	<LOD		
	Max	18.5	88.2	21.6	<LOD	<LOD	<LOD		

Note: ENR: enrofloxacin; CIP: ciprofloxacin; OFL: ofloxacin; PFLX: pefloxacin; NOR: norfloxacin; LOM: lomefloxacin. When minimum, maximum, and percentile data are not detected, they are displayed as <LOD to accurately represent the original result. In this study, viscera refers specifically to liver samples from livestock and poultry (22 pig livers, 5 chicken livers). If the Sample number does not reach 30, the data of detection rate and exceedance rate are for reference only; Only the checked QNs are shown in the table.

**Table 5:** Detection and exceedance rates of QNs across different food packaging types.

Food Category	Packaging Type	Sample Number	Detection Rate [% (*n*)]	Exceedance Rate [% (*n*)]
Total	In bulk	1293	9.13% (118)	2.47% (32)
	Pre-packaged	319	2.19% (7)	1.25% (4)
	*p*		<0.001	0.186
Chicken	In bulk	286	6.64% (19)	1.40% (4)
	Pre-packaged	54	7.40% (4)	1.85% (1)
	*p*		0.170	0.800
Eggs	In bulk	391	5.37% (21)	5.12% (20)
	Pre-packaged	205	1.46% (3)	1.46% (3)
	*p*		0.021	0.028
Fish	In bulk	178	35.39% (63)	3.37% (6)
	Pre-packaged	2	0% (0)	0% (0)
	*p*		0.297	0.792
Pork	In bulk	134	1.49% (2)	0.75% (1)
	Pre-packaged	1	0% (0)	0% (0)
	*p*		0.902	0.931
Crustaceans	In bulk	15	26.67% (4)	6.67% (1)
	Pre-packaged	-	-	-
Duck	In bulk	66	3.03% (2)	0% (0)
	Pre-packaged	-	-	-
Mollusk	In bulk	150	2.00% (3)	0% (0)
	Pre-packaged	-	-	-
Viscera	In bulk	27	14.81% (4)	0% (0)
	Pre-packaged	-	-	-

**Table 6:** Detection rate of different QNs in the six sampling sites.

Sampling Sites (*n* = Sampling Number)	Detection Rate [% (*n*)]					
	ENR	CIP	OFL	PFLX	NOR	LOM
Eatery (*n* = 68)	0% (0)	0% (0)	0% (0)	0% (0)	0% (0)	0% (0)
Farm (*n* = 82)	0% (0)	0% (0)	0% (0)	0% (0)	0% (0)	0% (0)
Farmers market (*n* = 784)	7.01% (50)	0.84% (6)	1.26% (9)	0.14% (1)	0.14% (1)	0% (0)
Fresh market (*n* = 219)	16.84% (33)	0.51% (1)	0% (0)	0% (0)	0% (0)	0% (0)
Living supermarket (*n* = 371)	4.31% (16)	0.54% (2)	1.35% (5)	0% (0)	0% (0)	0% (0)
Online store (*n* = 238)	5.15% (10)	0% (0)	0.51% (1)	0% (0)	0% (0)	0% (0)

## Data Availability

The original contributions presented in this study are included in the article. Further inquiries can be directed to the corresponding authors.

## Appendix A

**Table A1:** Sampling information for various foods from 2016 to 2023.

Sampling Year	Sales Outlets	Food Category	Sampling Number
2016	Farmers market	Chicken	16
		Duck	18
		Pork	20
		Viscera	12
	Living supermarket	Chicken	6
		Duck	4
		Pork	24
		Viscera	10
2017	Farmers market	Chicken	11
		Duck	11
		Eggs	20
	Living supermarket	Eggs	16
2018	Farmers market	Chicken	33
		Duck	33
		Eggs	30
	Living supermarket	Eggs	30
		Milk	55
2019	Farmers market	Chicken	34
		Eggs	41
		Viscera	5
	Fresh market	Eggs	3
	Living supermarket	Chicken	26
		Eggs	31
2020	Farmers market	Crustacean	4
		Eggs	18
		Fish	2
	Fresh market	Eggs	14
	Living supermarket	Crustacean	1
		Eggs	16
		Fish	3
	Online store	Eggs	12
2021	Eatery	Mollusk	7
	Farmers market	Chicken	6
		Eggs	27
		Fish	9
		Mollusk	23
		Pork	27
	Fresh market	Eggs	13
		Fish	18
		Pork	12
	Living supermarket	Chicken	6
		Eggs	22
		Fish	4
		Pork	4
	Online store	Chicken	8
		Eggs	18
		Fish	19
		Pork	12
2022	Eatery	Beef	8
		Mollusk	10
	Farm	Beef	7
	Farmers market	Chicken	30
		Eggs	24
		Fish	43
		Mollusk	50
	Fresh market	Beef	12
		Chicken	13
		Eggs	12
		Fish	20
		Pork	10
	Living supermarket	Chicken	17
		Eggs	23
		Fish	2
	Online store	Beef	3
		Chicken	15
		Eggs	16
		Pork	8
2023	Eatery	Beef	7
		Mollusk	24
	Farm	Eggs	75
	Farmers market	Chicken	48
		Crustacean	5
		Eggs	28
		Fish	49
		Mollusk	36
	Fresh market	Beef	6
		Chicken	25
		Crustacean	3
		Eggs	14
		Fish	11
		Pork	10
	Living supermarket	Chicken	23
		Crustacean	2
		Eggs	46
	Online store	Eggs	47
		Chicken	23
		Pork	8
		Beef	5

**Table A2:** Quinolone residues (P25–P95) detected in various foods from Guangzhou, 2016–2023 (unit: μg/kg).

Food Category (*n* = Sampling Number)	Statistic	ENR	CIP	OFL	PFLX	NOR	LOM
Total (*n* = 1612)	P25	<LOD	<LOD	<LOD	<LOD	<LOD	<LOD
	P50	<LOD	<LOD	<LOD	<LOD	<LOD	<LOD
	P75	<LOD	<LOD	<LOD	<LOD	<LOD	<LOD
	P95	1.933	<LOD	<LOD	<LOD	<LOD	<LOD
Beef (*n* = 48)	P25	<LOD	<LOD	<LOD	<LOD	<LOD	<LOD
	P50	<LOD	<LOD	<LOD	<LOD	<LOD	<LOD
	P75	<LOD	<LOD	<LOD	<LOD	<LOD	<LOD
	P95	<LOD	<LOD	<LOD	<LOD	<LOD	<LOD
Chicken (*n* = 340)	P25	<LOD	<LOD	<LOD	<LOD	<LOD	<LOD
	P50	<LOD	<LOD	<LOD	<LOD	<LOD	<LOD
	P75	<LOD	<LOD	<LOD	<LOD	<LOD	<LOD
	P95	<LOD	<LOD	<LOD	<LOD	<LOD	<LOD
Crustaceans (*n* = 15)	P25	<LOD	<LOD	<LOD	<LOD	<LOD	<LOD
	P50	<LOD	<LOD	<LOD	<LOD	<LOD	<LOD
	P75	2.71	<LOD	<LOD	<LOD	<LOD	<LOD
	P95	71.76	1.29	<LOD	<LOD	<LOD	<LOD
Duck (*n* = 66)	P25	<LOD	<LOD	<LOD	<LOD	<LOD	<LOD
	P50	<LOD	<LOD	<LOD	<LOD	<LOD	<LOD
	P75	<LOD	<LOD	<LOD	<LOD	<LOD	<LOD
	P95	<LOD	<LOD	<LOD	<LOD	<LOD	<LOD
Eggs (*n* = 596)	P25	<LOD	<LOD	<LOD	<LOD	<LOD	<LOD
	P50	<LOD	<LOD	<LOD	<LOD	<LOD	<LOD
	P75	<LOD	<LOD	<LOD	<LOD	<LOD	<LOD
	P95	<LOD	<LOD	<LOD	<LOD	<LOD	<LOD
Fish (*n* = 180)	P25	<LOD	<LOD	<LOD	<LOD	<LOD	<LOD
	P50	<LOD	<LOD	<LOD	<LOD	<LOD	<LOD
	P75	11.40	<LOD	<LOD	<LOD	<LOD	<LOD
	P95	69.71	<LOD	<LOD	<LOD	<LOD	<LOD
Milk (*n* = 55)	P25	<LOD	<LOD	<LOD	<LOD	<LOD	<LOD
	P50	<LOD	<LOD	<LOD	<LOD	<LOD	<LOD
	P75	<LOD	<LOD	<LOD	<LOD	<LOD	<LOD
	P95	<LOD	<LOD	<LOD	<LOD	<LOD	<LOD
Mollusk (*n* = 150)	P25	<LOD	<LOD	<LOD	<LOD	<LOD	<LOD
	P50	<LOD	<LOD	<LOD	<LOD	<LOD	<LOD
	P75	<LOD	<LOD	<LOD	<LOD	<LOD	<LOD
	P95	<LOD	<LOD	<LOD	<LOD	<LOD	<LOD
Pork (*n* = 135)	P25	<LOD	<LOD	<LOD	<LOD	<LOD	<LOD
	P50	<LOD	<LOD	<LOD	<LOD	<LOD	<LOD
	P75	<LOD	<LOD	<LOD	<LOD	<LOD	<LOD
	P95	<LOD	<LOD	<LOD	<LOD	<LOD	<LOD
Viscera (*n* = 27)	P25	<LOD	<LOD	<LOD	<LOD	<LOD	<LOD
	P50	<LOD	<LOD	<LOD	<LOD	<LOD	<LOD
	P75	<LOD	<LOD	<LOD	<LOD	<LOD	<LOD
	P95	11.35	4.865	<LOD	<LOD	<LOD	<LOD

Note: ENR: enrofloxacin; CIP: ciprofloxacin; OFL: ofloxacin; PFLX: pefloxacin; NOR: norfloxacin; LOM: lomefloxacin. When minimum, maximum, and percentile data are not detected, they are displayed as <LOD to accurately represent the original result. In this study, viscera refers specifically to liver samples from livestock and poultry (22 pig livers, 5 chicken livers). If the Sample number does not reach 30, the data of detection rate and exceedance rate are for reference only; Only the checked QNs are shown in the table.

**Table A3:** The average concentration, minimum concentration, and maximum concentration at sampling point QNs.

Sampling Sites		ENR	CIP	OFL	PFLX	NOR	LOM
Eatery (*n* = 68)	Mean	1.12	1.14	1.14	1.09	1.14	1.14
	SD	0.62	0.60	0.60	0.62	0.60	0.60
	Min	<LOD	<LOD	<LOD	<LOD	<LOD	<LOD
	Max	<LOD	<LOD	<LOD	<LOD	<LOD	<LOD
Farm (*n* = 82)	Mean	0.46	0.47	0.47	0.47	0.47	0.47
	SD	0.13	0.10	0.10	0.10	0.10	0.10
	Min	<LOD	<LOD	<LOD	<LOD	<LOD	<LOD
	Max	<LOD	<LOD	<LOD	<LOD	<LOD	<LOD
Farmers market (*n* = 784)	Mean	4.91	1.03	0.90	0.85	0.85	0.85
	SD	42.20	3.50	1.02	0.63	0.59	0.58
	Min	0.42	0.80	0.70	<LOD	<LOD	<LOD
	Max	1003.00	88.20	21.60	7.30	3.64	<LOD
Fresh market (*n* = 219)	Mean	8.10	0.81	0.80	0.77	0.80	0.80
	SD	33.56	0.67	0.65	0.64	0.65	0.65
	Min	0.35	<LOD	<LOD	<LOD	<LOD	<LOD
	Max	301.00	3.13	<LOD	<LOD	<LOD	<LOD
Living supermarket (*n* = 371)	Mean	2.96	0.72	2.43	0.62	0.63	0.63
	SD	39.98	1.33	33.68	0.47	0.48	0.48
	Min	0.37	12.50	1.39	<LOD	<LOD	<LOD
	Max	770.00	21.50	650.00	<LOD	<LOD	<LOD
Online store (*n* = 238)	Mean	1.85	0.66	0.81	0.61	0.66	0.66
	SD	8.01	0.61	2.14	0.58	0.61	0.61
	Min	0.30	<LOD	<LOD	<LOD	<LOD	<LOD
	Max	93.80	<LOD	29.40	<LOD	<LOD	<LOD

**Table A4:** Dietary consumption per reference person in Groups at varying ages (g/day).

Food Category	Groups at Varying Age (Years)				
	3–6	7–17	18–59	≥60	Total
Beef	9.17 ± 17.76	14.08 ± 23.24	16.82 ± 28.47	12.17 ± 27.83	15.35 ± 26.75
Chicken	24.50 ± 27.80	39.70 ± 39.80	43.62 ± 43.82	34.64 ± 31.14	40.61 ± 41.61
Crustaceans	2.98 ± 8.64	4.91 ± 13.57	6.25 ± 20.43	6.98 ± 17.83	5.75 ± 18.38
Duck	4.18 ± 9.94	7.58 ± 17.38	8.97 ± 20.67	7.73 ± 16.61	8.17 ± 19.13
Eggs	35.29 ± 36.69	33.80 ± 31.53	32.52 ± 30.44	37.85 ± 45.04	33.28 ± 32.15
Fish	27.03 ± 29.66	38.33 ± 35.94	46.11 ± 42.89	49.79 ± 46.48	43.13 ± 41.22
Milk	98.69 ± 111.41	85.74 ± 102.03	53.83 ± 79.02	44.27 ± 65.07	63.48 ± 88.14
Mollusk	0.80 ± 4.21	1.95 ± 7.80	2.22 ± 8.93	2.33 ± 8.04	2.05 ± 8.37
Pork	59.97 ± 43.24	88.35 ± 64.44	98.57 ± 69.90	94.71 ± 61.54	93.05 ± 67.39
Viscera	2.74 ± 9.87	4.78 ± 13.83	5.70 ± 16.72	6.57 ± 17.44	5.31 ± 15.76

**Table A5:** EDI of different QNs (ng/kg∙d) in various food categories (mean, 95%CI).

Age	QNs	Food Category										
		Beef	Chicken	Crustaceans	Duck	Eggs	Fish	Milk	Mollusk	Pork	Viscera	Total
3–6	ENR + CIP	1.09 × 10^−4^ (0–4.70 × 10^−4^)	9.67 × 10^−3^ (0–7.78 × 10^−3^)	2.36 × 10^−3^ (0–3.00 × 10^−2^)	1.96 × 10^−3^ (0–6.57 × 10^−4^)	1.38 × 10^−2^ (0–1.08 × 10^−2^)	3.28 × 10^−2^ (0–1.37 × 10^−1^)	4.93 × 10^−3^ (0–1.41 × 10^−2^)	5.44 × 10^−3^ (0–1.23 × 10^−3^)	1.37 × 10^−3^ (0–3.22 × 10^−3^)	9.70 × 10^−3^ (0–2.95 × 10^−3^)	1.36 × 10^−2^ (0, 4.99 × 10^−2^)
	OFL	6.88 × 10^−5^ (0–2.88 × 10^−4^)	1.63 × 10^−3^ (0–3.78 × 10^−3^)	7.45 × 10^−5^ (0–4.30 × 10^−4^)	6.15 × 10^−5^ (0–3.12 × 10^−4^)	3.99 × 10^−3^ (0–5.23 × 10^−3^)	1.99 × 10^−3^ (0–5.65 × 10^−3^)	2.47 × 10^−3^ (0–7.05 × 10^−3^)	6.02 × 10^−5^ (0–5.79 × 10^−4^)	6.95 × 10^−4^ (0–1.59 × 10^−3^)	1.51 × 10^−4^ (0–3.85 × 10^−4^)	2.56 × 10^−3^ (1.16 × 10^−4^, 3.22 × 10^−3^)
	PFLX	6.88 × 10^−5^ (0–2.88 × 10^−4^)	3.11 × 10^−2^ (0–3.70 × 10^−3^)	7.45 × 10^−5^ (0–4.30 × 10^−4^)	6.16 × 10^−5^ (0–3.15 × 10^−4^)	1.22 × 10^−3^ (0–4.97 × 10^−3^)	1.98 × 10^−3^ (0–5.64 × 10^−3^)	2.47 × 10^−3^ (0–7.05 × 10^−3^)	6.02 × 10^−5^ (0–5.79 × 10^−4^)	8.49 × 10^−4^ (0–2.11 × 10^−3^)	4.51 × 10^−5^ (0–3.34 × 10^−4^)	2.40 × 10^−3^ (5.39 × 10^−5^, 1.10 × 10^−3^)
	NOR	6.88 × 10^−5^ (0–2.88 × 10^−4^)	7.96 × 10^−4^ (0–3.67 × 10^−3^)	7.45 × 10^−5^ (0–4.30 × 10^−4^)	6.16 × 10^−5^ (0–3.14 × 10^−4^)	1.22 × 10^−3^ (0–4.90 × 10^−3^)	1.99 × 10^−3^ (0–5.66 × 10^−3^)	2.47 × 10^−3^ (0–7.05 × 10^−3^)	6.02 × 10^−5^ (0–5.79 × 10^−4^)	7.67 × 10^−4^ (0–1.68 × 10^−3^)	4.47 × 10^−5^ (0–3.28 × 10^−4^)	5.43 × 10^−4^ (4.84 × 10^−5^, 1.08 × 10^−3^)
	LOM	6.88 × 10^−5^ (0–2.88 × 10^−4^)	7.79 × 10^−4^ (0–3.60 × 10^−3^)	7.45 × 10^−5^ (0–4.30 × 10^−4^)	6.07 × 10^−5^ (0–3.10 × 10^−4^)	1.22 × 10^−3^ (0–4.97 × 10^−3^)	1.98 × 10^−3^ (0–5.65 × 10^−3^)	2.47 × 10^−3^ (0–7.05 × 10^−3^)	6.02 × 10^−5^ (0–5.79 × 10^−4^)	7.01 × 10^−4^ (0–1.61 × 10^−3^)	4.52 × 10^−5^ (0–3.29 × 10^−4^)	3.01 × 10^−4^ (2.85 × 10^−5^, 6.00 × 10^−4^)
7–17	ENR + CIP	8.33 × 10^−5^ (0–3.19 × 10^−4^)	9.64 × 10^−3^ (0–5.84 × 10^−3^)	2.33 × 10^−3^ (0–2.54 × 10^−2^)	1.09 × 10^−3^ (0–5.77 × 10^−4^)	8.41 × 10^−3^ (0–4.82 × 10^−3^)	2.62 × 10^−2^ (0–9.23 × 10^−2^)	2.14 × 10^−3^ (0–6.34 × 10^−3^)	5.90 × 10^−3^ (0–1.18 × 10^−3^)	1.00 × 10^−3^ (0–2.43 × 10^−3^)	1.11 × 10^−2^ (0–2.61 × 10^−3^)	1.03 × 10^−2^ (0, 3.55 × 10^−2^)
	OFL	5.28 × 10^−5^ (0–1.96 × 10^−4^)	1.02 × 10^−3^ (0–2.84 × 10^−3^)	6.13 × 10^−5^ (0–3.40 × 10^−4^)	5.58 × 10^−5^ (0–2.77 × 10^−4^)	1.57 × 10^−3^ (0–2.34 × 10^−3^)	1.41 × 10^−3^ (0–3.63 × 10^−3^)	1.07 × 10^−3^ (0–3.17 × 10^−3^)	7.31 × 10^−5^ (0–5.54 × 10^−4^)	5.19 × 10^−4^ (0–1.18 × 10^−3^)	1.38 × 10^−4^ (0–2.75 × 10^−4^)	1.53 × 10^−3^ (1.28 × 10^−4^, 1.85 × 10^−3^)
	PFLX	5.28 × 10^−5^ (0–1.96 × 10^−4^)	2.63 × 10^−2^ (0–2.86 × 10^−3^)	6.13 × 10^−5^ (0–3.40 × 10^−4^)	5.59 × 10^−5^ (0–2.74 × 10^−4^)	5.83 × 10^−4^ (0–2.24 × 10^−3^)	1.40 × 10^−3^ (0–3.63 × 10^−3^)	1.07 × 10^−3^ (0–3.17 × 10^−3^)	7.31 × 10^−5^ (0–5.54 × 10^−4^)	6.25 × 10^−4^ (0–1.52 × 10^−3^)	3.94 × 10^−5^ (0–2.42 × 10^−4^)	1.75 × 10^−3^ (5.60 × 10^−5^, 6.36 × 10^−4^)
	NOR	5.28 × 10^−5^ (0–1.96 × 10^−4^)	6.32 × 10^−4^ (0–2.72 × 10^−3^)	6.13 × 10^−5^ (0–3.40 × 10^−4^)	5.50 × 10^−5^ (0–2.72 × 10^−4^)	5.82 × 10^−4^ (0–2.22 × 10^−3^)	1.41 × 10^−3^ (0–3.63 × 10^−3^)	1.07 × 10^−3^ (0–3.17 × 10^−3^)	7.31 × 10^−5^ (0–5.54 × 10^−4^)	5.72 × 10^−4^ (0–1.23 × 10^−3^)	3.93 × 10^−5^ (0–2.44 × 10^−4^)	3.30 × 10^−4^ (4.95 × 10^−5^, 6.33 × 10^−4^)
	LOM	5.28 × 10^−5^ (0–1.96 × 10^−4^)	6.35 × 10^−4^ (0–2.78 × 10^−3^)	6.13 × 10^−5^ (0–3.40 × 10^−4^)	5.52 × 10^−5^ (0–2.71 × 10^−4^)	5.94 × 10^−4^ (0–2.25 × 10^−3^)	1.41 × 10^−3^ (0–3.64 × 10^−3^)	1.07 × 10^−3^ (0–3.17 × 10^−3^)	7.31 × 10^−5^ (0–5.54 × 10^−4^)	5.15 × 10^−4^ (0–1.18 × 10^−3^)	3.85 × 10^−5^ (0–2.41 × 10^−4^)	1.82 × 10^−4^ (3.04 × 10^−5^, 3.47 × 10^−4^)
18–59	ENR + CIP	6.45 × 10^−5^ (0–2.49 × 10^−4^)	6.20 × 10^−3^ (0–4.09 × 10^−3^)	1.94 × 10^−3^ (0–2.20 × 10^−2^)	2.54 × 10^−3^ (0–4.50 × 10^−4^)	3.26 × 10^−3^ (0–3.00 × 10^−3^)	1.93 × 10^−2^ (0–7.19 × 10^−2^)	8.68 × 10^−4^ (0–2.96 × 10^−3^)	3.91 × 10^−3^ (0–8.68 × 10^−4^)	7.07 × 10^−4^ (0–1.70 × 10^−3^)	8.96 × 10^−3^ (0–1.64 × 10^−3^)	8.18 × 10^−3^ (0, 3.03 × 10^−2^)
	OFL	4.07 × 10^−5^ (0–1.54 × 10^−4^)	6.71 × 10^−4^ (0–2.05 × 10^−3^)	5.04 × 10^−5^ (0–3.21 × 10^−4^)	4.24 × 10^−5^ (0–2.09 × 10^−4^)	1.05 × 10^−3^ (0–1.48 × 10^−3^)	1.09 × 10^−3^ (0–2.80 × 10^−3^)	4.34 × 10^−4^ (0–1.48 × 10^−3^)	5.37 × 10^−5^ (0–4.09 × 10^−4^)	3.72 × 10^−4^ (0–8.30 × 10^−4^)	8.97 × 10^−5^ (0–2.17 × 10^−4^)	8.62 × 10^−4^ (6.73 × 10^−5^, 1.25 × 10^−3^)
	PFLX	4.07 × 10^−5^ (0–1.54 × 10^−4^)	1.51 × 10^−2^ (0–1.97 × 10^−3^)	5.04 × 10^−5^ (0–3.21 × 10^−4^)	4.21 × 10^−5^ (0–2.11 × 10^−4^)	3.66 × 10^−4^ (0–1.42 × 10^−3^)	1.09 × 10^−3^ (0–2.81 × 10^−3^)	4.34 × 10^−4^ (0–1.48 × 10^−3^)	5.37 × 10^−5^ (0–4.09 × 10^−4^)	4.49 × 10^−4^ (0–1.09 × 10^−3^)	2.96 × 10^−5^ (0–1.86 × 10^−4^)	1.14 × 10^−3^ (3.49 × 10^−5^, 4.28 × 10^−4^)
	NOR	4.07 × 10^−5^ (0–1.54 × 10^−4^)	4.53 × 10^−4^ (0–1.98 × 10^−3^)	5.04 × 10^−5^ (0–3.21 × 10^−4^)	4.24 × 10^−5^ (0–2.08 × 10^−4^)	3.62 × 10^−4^ (0–1.39 × 10^−3^)	1.10 × 10^−3^ (0–2.81 × 10^−3^)	4.34 × 10^−4^ (0–1.48 × 10^−3^)	5.37 × 10^−5^ (0–4.09 × 10^−4^)	4.08 × 10^−4^ (0–8.80 × 10^−4^)	3.06 × 10^−5^ (0–1.94 × 10^−4^)	2.13 × 10^−4^ (3.19 × 10^−5^, 4.20 × 10^−4^)
	LOM	4.07 × 10^−5^ (0–1.54 × 10^−4^)	4.53 × 10^−4^ (0–1.93 × 10^−3^)	5.04 × 10^−5^ (0–3.21 × 10^−4^)	4.22 × 10^−5^ (0–2.10 × 10^−4^)	3.64 × 10^−4^ (0–1.41 × 10^−3^)	1.09 × 10^−3^ (0–2.81 × 10^−3^)	4.34 × 10^−4^ (0–1.48 × 10^−3^)	5.37 × 10^−5^ (0–4.09 × 10^−4^)	3.77 × 10^−4^ (0–8.44 × 10^−4^)	3.04 × 10^−5^ (0–1.89 × 10^−4^)	1.18 × 10^−4^ (1.76 × 10^−5^, 2.29 × 10^−4^)
≥60	ENR + CIP	4.83 × 10^−5^ (0–2.36 × 10^−4^)	4.01 × 10^−3^ (0–3.18 × 10^−3^)	2.00 × 10^−3^ (0–2.16 × 10^−2^)	2.86 × 10^−3^ (0–3.78 × 10^−4^)	3.41 × 10^−3^ (0–4.16 × 10^−3^)	2.34 × 10^−2^ (0–8.29 × 10^−2^)	7.38 × 10^−4^ (0–2.52 × 10^−3^)	5.03 × 10^−3^ (0–8.26 × 10^−4^)	7.22 × 10^−4^ (0–1.63 × 10^−3^)	7.97 × 10^−3^ (0–2.22 × 10^−3^)	8.96 × 10^−3^ (0, 3.29 × 10^−2^)
	OFL	3.04 × 10^−5^ (0–1.45 × 10^−4^)	6.28 × 10^−4^ (0–1.54 × 10^−3^)	5.82 × 10^−5^ (0–3.02 × 10^−4^)	3.76 × 10^−5^ (0–1.75 × 10^−4^)	1.13 × 10^−3^ (0–2.03 × 10^−3^)	1.22 × 10^−3^ (0–3.13 × 10^−3^)	3.69 × 10^−4^ (0–1.26 × 10^−3^)	5.82 × 10^−5^ (0–3.89 × 10^−4^)	3.68 × 10^−4^ (0–7.93 × 10^−4^)	1.13 × 10^−4^ (0–2.44 × 10^−4^)	9.62 × 10^−4^ (6.28 × 10^−5^, 1.30 × 10^−3^)
	PFLX	3.04 × 10^−5^ (0–1.45 × 10^−4^)	1.01 × 10^−2^ (0–1.55 × 10^−3^)	5.82 × 10^−5^ (0–3.02 × 10^−4^)	3.80 × 10^−5^ (0–1.78 × 10^−4^)	4.36 × 10^−4^ (0–1.90 × 10^−3^)	1.21 × 10^−3^ (0–3.14 × 10^−3^)	3.69 × 10^−4^ (0–1.26 × 10^−3^)	5.82 × 10^−5^ (0–3.89 × 10^−4^)	4.47 × 10^−4^ (0–1.07 × 10^−3^)	3.62 × 10^−5^ (0–2.05 × 10^−4^)	1.22 × 10^−3^ (3.46 × 10^−5^, 4.34 × 10^−4^)
	NOR	3.04 × 10^−5^ (0–1.45 × 10^−4^)	3.70 × 10^−4^ (0–1.50 × 10^−3^)	5.82 × 10^−5^ (0–3.02 × 10^−4^)	3.76 × 10^−5^ (0–1.77 × 10^−4^)	4.32 × 10^−4^ (0–1.85 × 10^−3^)	1.22 × 10^−3^ (0–3.14 × 10^−3^)	3.69 × 10^−4^ (0–1.26 × 10^−3^)	5.82 × 10^−5^ (0–3.89 × 10^−4^)	4.09 × 10^−4^ (0–8.37 × 10^−4^)	3.66 × 10^−5^ (0–2.10 × 10^−4^)	2.18 × 10^−4^ (3.14 × 10^−5^, 4.23 × 10^−4^)
	LOM	3.04 × 10^−5^ (0–1.45 × 10^−4^)	3.67 × 10^−4^ (0–1.50 × 10^−3^)	5.82 × 10^−5^ (0–3.02 × 10^−4^)	3.79 × 10^−5^ (0–1.78 × 10^−4^)	4.40 × 10^−4^ (0–1.94 × 10^−3^)	1.22 × 10^−3^ (0–3.14 × 10^−3^)	3.69 × 10^−4^ (0–1.26 × 10^−3^)	5.82 × 10^−5^ (0–3.89 × 10^−4^)	3.67 × 10^−4^ (0–7.99 × 10^−4^)	3.64 × 10^−5^ (0–2.09 × 10^−4^)	1.20 × 10^−4^ (1.81 × 10^−5^, 2.34 × 10^−4^)
Total	ENR + CIP	6.05 × 10^−5^ (0–2.43 × 10^−4^)	4.58 × 10^−3^ (0–4.02 × 10^−3^)	1.54 × 10^−3^ (0–1.97 × 10^−2^)	1.66 × 10^−3^ (0–4.30 × 10^−4^)	3.58 × 10^−3^ (0–3.25 × 10^−3^)	1.82 × 10^−2^ (0–7.08 × 10^−2^)	1.06 × 10^−3^ (0–3.47 × 10^−3^)	6.06 × 10^−3^ (0–8.51 × 10^−4^)	7.07 × 10^−4^ (0–1.70 × 10^−3^)	8.23 × 10^−3^ (0–1.96 × 10^−3^)	7.34 × 10^−3^ (0, 2.93 × 10^−2^)
	OFL	3.84 × 10^−5^ (0–1.48 × 10^−4^)	8.51 × 10^−4^ (0–1.96 × 10^−3^)	4.79 × 10^−5^ (0–3.00 × 10^−4^)	3.92 × 10^−5^ (0–1.97 × 10^−4^)	1.21 × 10^−3^ (0–1.56 × 10^−3^)	1.06 × 10^−3^ (0–2.75 × 10^−3^)	5.29 × 10^−4^ (0–1.74 × 10^−3^)	5.12 × 10^−5^ (0–3.95 × 10^−4^)	3.63 × 10^−4^ (0–8.22 × 10^−4^)	9.66 × 10^−5^ (0–2.10 × 10^−4^)	9.46 × 10^−4^ (7.76 × 10^−5^, 1.26 × 10^−3^)
	PFLX	3.84 × 10^−5^ (0–1.48 × 10^−4^)	1.35 × 10^−2^ (0–1.93 × 10^−3^)	4.79 × 10^−5^ (0–3.00 × 10^−4^)	3.96 × 10^−5^ (0–1.99 × 10^−4^)	3.82 × 10^−4^ (0–1.50 × 10^−3^)	1.05 × 10^−3^ (0–2.76 × 10^−3^)	5.29 × 10^−4^ (0–1.74 × 10^−3^)	5.12 × 10^−5^ (0–3.95 × 10^−4^)	4.33 × 10^−4^ (0–1.03 × 10^−3^)	2.91 × 10^−5^ (0–1.83 × 10^−4^)	1.45 × 10^−3^ (3.23 × 10^−5^, 4.32 × 10^−4^)
	NOR	3.84 × 10^−5^ (0–1.48 × 10^−4^)	4.42 × 10^−4^ (0–1.94 × 10^−3^)	4.79 × 10^−5^ (0–3.00 × 10^−4^)	3.98 × 10^−5^ (0–2.00 × 10^−4^)	3.82 × 10^−4^ (0–1.51 × 10^−3^)	1.06 × 10^−3^ (0–2.76 × 10^−3^)	5.29 × 10^−4^ (0–1.74 × 10^−3^)	5.12 × 10^−5^ (0–3.95 × 10^−4^)	4.02 × 10^−4^ (0–8.84 × 10^−4^)	2.90 × 10^−5^ (0–1.82 × 10^−4^)	2.17 × 10^−4^ (3.13 × 10^−5^, 4.24 × 10^−4^)
	LOM	3.84 × 10^−5^ (0–1.48 × 10^−4^)	4.40 × 10^−4^ (0–1.94 × 10^−3^)	4.79 × 10^−5^ (0–3.00 × 10^−4^)	3.97 × 10^−5^ (0–2.00 × 10^−4^)	3.81 × 10^−4^ (0–1.48 × 10^−3^)	1.06 × 10^−3^ (0–2.76 × 10^−3^)	5.29 × 10^−4^ (0–1.74 × 10^−3^)	5.12 × 10^−5^ (0–3.95 × 10^−4^)	3.60 × 10^−4^ (0–8.23 × 10^−4^)	2.95 × 10^−5^ (0–1.85 × 10^−4^)	1.19 × 10^−4^ (1.97 × 10^−5^, 2.32 × 10^−4^)

ENR: enrofloxacin; CIP: ciprofloxacin; OFL: ofloxacin; PFLX: pefloxacin; NOR: norfloxacin; LOM: lomefloxacin.

**Table A6:** HQ of different QNs in various food categories (mean, 95%CI).

Age	QNs	Food Category										
		Beef	Chicken	Crustaceans	Duck	Eggs	Fish	Milk	Mollusk	Pork	Viscera	Total
3–6	ENR + CIP	1.75 × 10^−5^ (0, 7.58 × 10^−5^)	1.56 × 10^−3^ (0, 1.26 × 10^−3^)	3.81 × 10^−4^ (0, 4.84 × 10^−3^)	3.16 × 10^−4^ (0, 1.06 × 10^−4^)	2.23 × 10^−3^ (0, 1.75 × 10^−3^)	5.28 × 10^−3^ (0, 2.20 × 10^−2^)	7.96 × 10^−4^ (0, 2.27 × 10^−3^)	8.78 × 10^−4^ (0, 1.99 × 10^−4^)	1.37 × 10^−3^ (0, 3.22 × 10^−3^)	1.56 × 10^−3^ (0, 4.76 × 10^−4^)	1.36 × 10^−2^ (0, 4.99 × 10^−2^)
	OFL	1.38 × 10^−5^ (0, 5.76 × 10^−5^)	3.26 × 10^−4^ (0, 7.56 × 10^−4^)	1.49 × 10^−5^ (0, 8.59 × 10^−5^)	1.23 × 10^−5^ (0, 6.25 × 10^−5^)	7.97 × 10^−4^ (0, 1.05 × 10^−3^)	3.97 × 10^−4^ (0, 1.13 × 10^−3^)	4.93 × 10^−4^ (0, 1.41 × 10^−3^)	1.20 × 10^−5^ (0, 1.16 × 10^−4^)	6.95 × 10^−4^ (0, 1.59 × 10^−3^)	3.02 × 10^−5^ (0, 7.71 × 10^−5^)	2.56 × 10^−3^ (1.16 × 10^−4^, 3.22 × 10^−3^)
	PFLX	4.91 × 10^−6^ (0, 2.06 × 10^−5^)	2.22 × 10^−3^ (0, 2.64 × 10^−4^)	5.32 × 10^−6^ (0, 3.07 × 10^−5^)	4.40 × 10^−6^ (0, 2.25 × 10^−5^)	8.72 × 10^−5^ (0, 3.55 × 10^−4^)	1.41 × 10^−4^ (0, 4.03 × 10^−4^)	1.76 × 10^−4^ (0, 5.03 × 10^−4^)	4.30 × 10^−6^ (0, 4.14 × 10^−5^)	8.49 × 10^−4^ (0, 2.11 × 10^−3^)	3.22 × 10^−6^ (0, 2.39 × 10^−5^)	2.40 × 10^−3^ (5.39 × 10^−5^, 1.10 × 10^−3^)
	NOR	4.91 × 10^−6^ (0, 2.06 × 10^−5^)	5.69 × 10^−5^ (0, 2.62 × 10^−4^)	5.32 × 10^−6^ (0, 3.07 × 10^−5^)	4.40 × 10^−6^ (0, 2.25 × 10^−5^)	8.72 × 10^−5^ (0, 3.50 × 10^−4^)	1.42 × 10^−4^ (0, 4.04 × 10^−4^)	1.76 × 10^−4^ (0, 5.03 × 10^−4^)	4.30 × 10^−6^ (0, 4.14 × 10^−5^)	7.67 × 10^−4^ (0, 1.68 × 10^−3^)	3.19 × 10^−6^ (0, 2.34 × 10^−5^)	5.43 × 10^−4^ (4.84 × 10^−5^, 1.08 × 10^−3^)
	LOM	2.75 × 10^−6^ (0, 1.15 × 10^−5^)	3.12 × 10^−5^ (0, 1.44 × 10^−4^)	2.98 × 10^−6^ (0, 1.72 × 10^−5^)	2.43 × 10^−6^ (0, 1.24 × 10^−5^)	4.86 × 10^−5^ (0, 1.99 × 10^−4^)	7.94 × 10^−5^ (0, 2.26 × 10^−4^)	9.87 × 10^−5^ (0, 2.82 × 10^−4^)	2.41 × 10^−6^ (0, 2.32 × 10^−5^)	7.01 × 10^−4^ (0, 1.61 × 10^−3^)	1.81 × 10^−6^ (0, 1.32 × 10^−5^)	3.01 × 10^−4^ (2.85 × 10^−5^, 6.00 × 10^−4^)
7–17	ENR + CIP	1.34 × 10^−5^ (0, 5.14 × 10^−5^)	1.55 × 10^−3^ (0, 9.41 × 10^−4^)	3.76 × 10^−4^ (0, 4.10 × 10^−3^)	1.75 × 10^−4^ (0, 9.30 × 10^−5^)	1.36 × 10^−3^ (0, 7.77 × 10^−4^)	4.23 × 10^−3^ (0, 1.49 × 10^−2^)	3.46 × 10^−4^ (0, 1.02 × 10^−3^)	9.51 × 10^−4^ (0, 1.91 × 10^−4^)	1.00 × 10^−3^ (0, 2.43 × 10^−3^)	1.79 × 10^−3^ (0, 4.20 × 10^−4^)	1.03 × 10^−2^ (0, 3.55 × 10^−2^)
	OFL	1.06 × 10^−5^ (0, 3.92 × 10^−5^)	2.04 × 10^−4^ (0, 5.68 × 10^−4^)	1.23 × 10^−5^ (0, 6.80 × 10^−5^)	1.12 × 10^−5^ (0, 5.54 × 10^−5^)	3.13 × 10^−4^ (0, 4.67 × 10^−4^)	2.82 × 10^−4^ (0, 7.27 × 10^−4^)	2.14 × 10^−4^ (0, 6.34 × 10^−4^)	1.46 × 10^−5^ (0, 1.11 × 10^−4^)	5.19 × 10^−4^ (0, 1.18 × 10^−3^)	2.76 × 10^−5^ (0, 5.49 × 10^−5^)	1.53 × 10^−3^ (1.28 × 10^−4^, 1.85 × 10^−3^)
	PFLX	3.77 × 10^−6^ (0, 1.40 × 10^−5^)	1.88 × 10^−3^ (0, 2.04 × 10^−4^)	4.38 × 10^−6^ (0, 2.43 × 10^−5^)	3.99 × 10^−6^ (0, 1.96 × 10^−5^)	4.16 × 10^−5^ (0, 1.60 × 10^−4^)	1.00 × 10^−4^ (0, 2.59 × 10^−4^)	7.66 × 10^−5^ (0, 2.26 × 10^−4^)	5.22 × 10^−6^ (0, 3.96 × 10^−5^)	6.25 × 10^−4^ (0, 1.52 × 10^−3^)	2.81 × 10^−6^ (0, 1.73 × 10^−5^)	1.75 × 10^−3^ (5.60 × 10^−5^, 6.36 × 10^−4^)
	NOR	3.77 × 10^−6^ (0, 1.40 × 10^−5^)	4.51 × 10^−5^ (0, 1.94 × 10^−4^)	4.38 × 10^−6^ (0, 2.43 × 10^−5^)	3.93 × 10^−6^ (0, 1.94 × 10^−5^)	4.16 × 10^−5^ (0, 1.59 × 10^−4^)	1.01 × 10^−4^ (0, 2.59 × 10^−4^)	7.66 × 10^−5^ (0, 2.26 × 10^−4^)	5.22 × 10^−6^ (0, 3.96 × 10^−5^)	5.72 × 10^−4^ (0, 1.23 × 10^−3^)	2.80 × 10^−6^ (0, 1.74 × 10^−5^)	3.30 × 10^−4^ (4.95 × 10^−5^, 6.33 × 10^−4^)
	LOM	2.11 × 10^−6^ (0, 7.84 × 10^−6^)	2.54 × 10^−5^ (0, 1.11 × 10^−4^)	2.45 × 10^−6^ (0, 1.36 × 10^−5^)	2.21 × 10^−6^ (0, 1.09 × 10^−5^)	2.38 × 10^−5^ (0, 9.00 × 10^−5^)	5.65 × 10^−5^ (0, 1.45 × 10^−4^)	4.29 × 10^−5^ (0, 1.27 × 10^−4^)	2.92 × 10^−6^ (0, 2.22 × 10^−5^)	5.15 × 10^−4^ (0, 1.18 × 10^−3^)	1.54 × 10^−6^ (0, 9.65 × 10^−6^)	1.82 × 10^−4^ (3.04 × 10^−5^, 3.47 × 10^−4^)
18–59	ENR + CIP	1.04 × 10^−5^ (0, 4.02 × 10^−5^)	1.00 × 10^−3^ (0, 6.60 × 10^−4^)	3.12 × 10^−4^ (0, 3.55 × 10^−3^)	4.10 × 10^−4^ (0, 7.26 × 10^−5^)	5.26 × 10^−4^ (0, 4.84 × 10^−4^)	3.11 × 10^−3^ (0, 1.16 × 10^−2^)	1.40 × 10^−4^ (0, 4.78 × 10^−4^)	6.30 × 10^−4^ (0, 1.40 × 10^−4^)	7.07 × 10^−4^ (0, 1.70 × 10^−3^)	1.44 × 10^−3^ (0, 2.65 × 10^−4^)	8.18 × 10^−3^ (0, 3.03 × 10^−2^)
	OFL	8.14 × 10^−6^ (0, 3.08 × 10^−5^)	1.34 × 10^−4^ (0, 4.10 × 10^−4^)	1.01 × 10^−5^ (0, 6.43 × 10^−5^)	8.47 × 10^−6^ (0, 4.18 × 10^−5^)	2.11 × 10^−4^ (0, 2.96 × 10^−4^)	2.19 × 10^−4^ (0, 5.60 × 10^−4^)	8.68 × 10^−5^ (0, 2.96 × 10^−4^)	1.07 × 10^−5^ (0, 8.17 × 10^−5^)	3.72 × 10^−4^ (0, 8.30 × 10^−4^)	1.79 × 10^−5^ (0, 4.34 × 10^−5^)	8.62 × 10^−4^ (6.73 × 10^−5^, 1.25 × 10^−3^)
	PFLX	2.91 × 10^−6^ (0, 1.10 × 10^−5^)	1.08 × 10^−3^ (0, 1.41 × 10^−4^)	3.60 × 10^−6^ (0, 2.30 × 10^−5^)	3.01 × 10^−6^ (0, 1.51 × 10^−5^)	2.62 × 10^−5^ (0, 1.01 × 10^−4^)	7.76 × 10^−5^ (0, 2.00 × 10^−4^)	3.10 × 10^−5^ (0, 1.06 × 10^−4^)	3.83 × 10^−6^ (0, 2.92 × 10^−5^)	4.49 × 10^−4^ (0, 1.09 × 10^−3^)	2.11 × 10^−6^ (0, 1.33 × 10^−5^)	1.14 × 10^−3^ (3.49 × 10^−5^, 4.28 × 10^−4^)
	NOR	2.91 × 10^−6^ (0, 1.10 × 10^−5^)	3.24 × 10^−5^ (0, 1.42 × 10^−4^)	3.60 × 10^−6^ (0, 2.30 × 10^−5^)	3.03 × 10^−6^ (0, 1.49 × 10^−5^)	2.59 × 10^−5^ (0, 9.96 × 10^−5^)	7.82 × 10^−5^ (0, 2.01 × 10^−4^)	3.10 × 10^−5^ (0, 1.06 × 10^−4^)	3.83 × 10^−6^ (0, 2.92 × 10^−5^)	4.08 × 10^−4^ (0, 8.80 × 10^−4^)	2.19 × 10^−6^ (0, 1.39 × 10^−5^)	2.13 × 10^−4^ (3.19 × 10^−5^, 4.20 × 10^−4^)
	LOM	1.63 × 10^−6^ (0, 6.16 × 10^−6^)	1.81 × 10^−5^ (0, 7.72 × 10^−5^)	2.02 × 10^−6^ (0, 1.29 × 10^−5^)	1.69 × 10^−6^ (0, 8.42 × 10^−6^)	1.46 × 10^−5^ (0, 5.65 × 10^−5^)	4.38 × 10^−5^ (0, 1.12 × 10^−4^)	1.74 × 10^−5^ (0, 5.93 × 10^−5^)	2.15 × 10^−6^ (0, 1.63 × 10^−5^)	3.77 × 10^−4^ (0, 8.44 × 10^−4^)	1.21 × 10^−6^ (0, 7.58 × 10^−6^)	1.18 × 10^−4^ (1.76 × 10^−5^, 2.29 × 10^−4^)
≥60	ENR + CIP	7.79 × 10^−6^ (0, 3.81 × 10^−5^)	6.47 × 10^−4^ (0, 5.12 × 10^−4^)	3.22 × 10^−4^ (0, 3.49 × 10^−3^)	4.61 × 10^−4^ (0, 6.09 × 10^−5^)	5.51 × 10^−4^ (0, 6.71 × 10^−4^)	3.78 × 10^−3^ (0, 1.34 × 10^−2^)	1.19 × 10^−4^ (0, 4.07 × 10^−4^)	8.11 × 10^−4^ (0, 1.33 × 10^−4^)	7.22 × 10^−4^ (0, 1.63 × 10^−3^)	1.29 × 10^−3^ (0, 3.58 × 10^−4^)	8.96 × 10^−3^ (0, 3.29 × 10^−2^)
	OFL	6.08 × 10^−6^ (0, 2.90 × 10^−5^)	1.26 × 10^−4^ (0, 3.07 × 10^−4^)	1.16 × 10^−5^ (0, 6.05 × 10^−5^)	7.52 × 10^−6^ (0, 3.51 × 10^−5^)	2.25 × 10^−4^ (0, 4.05 × 10^−4^)	2.44 × 10^−4^ (0, 6.26 × 10^−4^)	7.38 × 10^−5^ (0, 2.52 × 10^−4^)	1.16 × 10^−5^ (0, 7.77 × 10^−5^)	3.68 × 10^−4^ (0, 7.93 × 10^−4^)	2.27 × 10^−5^ (0, 4.88 × 10^−5^)	9.62 × 10^−4^ (6.28 × 10^−5^, 1.30 × 10^−3^)
	PFLX	2.17 × 10^−6^ (0, 1.03 × 10^−5^)	7.19 × 10^−4^ (0, 1.11 × 10^−4^)	4.15 × 10^−6^ (0, 2.16 × 10^−5^)	2.71 × 10^−6^ (0, 1.27 × 10^−5^)	3.11 × 10^−5^ (0, 1.36 × 10^−4^)	8.66 × 10^−5^ (0, 2.24 × 10^−4^)	2.63 × 10^−5^ (0, 9.00 × 10^−5^)	4.16 × 10^−6^ (0, 2.78 × 10^−5^)	4.47 × 10^−4^ (0, 1.07 × 10^−3^)	2.59 × 10^−6^ (0, 1.47 × 10^−5^)	1.22 × 10^−3^ (3.46 × 10^−5^, 4.34 × 10^−4^)
	NOR	2.17 × 10^−6^ (0, 1.03 × 10^−5^)	2.64 × 10^−5^ (0, 1.07 × 10^−4^)	4.15 × 10^−6^ (0, 2.16 × 10^−5^)	2.69 × 10^−6^ (0, 1.26 × 10^−5^)	3.08 × 10^−5^ (0, 1.32 × 10^−4^)	8.72 × 10^−5^ (0, 2.24 × 10^−4^)	2.63 × 10^−5^ (0, 9.00 × 10^−5^)	4.16 × 10^−6^ (0, 2.78 × 10^−5^)	4.09 × 10^−4^ (0, 8.37 × 10^−4^)	2.62 × 10^−6^ (0, 1.50 × 10^−5^)	2.18 × 10^−4^ (3.14 × 10^−5^, 4.23 × 10^−4^)
	LOM	1.22 × 10^−6^ (0, 5.79 × 10^−6^)	1.47 × 10^−5^ (0, 6.01 × 10^−5^)	2.33 × 10^−6^ (0, 1.21 × 10^−5^)	1.52 × 10^−6^ (0, 7.12 × 10^−6^)	1.76 × 10^−5^ (0, 7.75 × 10^−5^)	4.88 × 10^−5^ (0, 1.26 × 10^−4^)	1.48 × 10^−5^ (0, 5.04 × 10^−5^)	2.33 × 10^−6^ (0, 1.55 × 10^−5^)	3.67 × 10^−4^ (0, 7.99 × 10^−4^)	1.46 × 10^−6^ (0, 8.36 × 10^−6^)	1.20 × 10^−4^ (1.81 × 10^−5^, 2.34 × 10^−4^)
Total	ENR + CIP	9.76 × 10^−6^ (0, 3.92 × 10^−5^)	7.38 × 10^−4^ (0, 6.48 × 10^−4^)	2.49 × 10^−4^ (0, 3.18 × 10^−3^)	2.67 × 10^−4^ (0, 6.94 × 10^−5^)	5.77 × 10^−4^ (0, 5.24 × 10^−4^)	2.93 × 10^−3^ (0, 1.14 × 10^−2^)	1.71 × 10^−4^ (0, 5.60 × 10^−4^)	9.78 × 10^−4^ (0, 1.37 × 10^−4^)	7.07 × 10^−4^ (0, 1.70 × 10^−3^)	1.33 × 10^−3^ (0, 3.17 × 10^−4^)	7.34 × 10^−3^ (0, 2.93 × 10^−2^)
	OFL	7.67 × 10^−6^ (0, 2.97 × 10^−5^)	1.70 × 10^−4^ (0, 3.91 × 10^−4^)	9.58 × 10^−6^ (0, 6.00 × 10^−5^)	7.84 × 10^−6^ (0, 3.93 × 10^−5^)	2.42 × 10^−4^ (0, 3.12 × 10^−4^)	2.11 × 10^−4^ (0, 5.50 × 10^−4^)	1.06 × 10^−4^ (0, 3.47 × 10^−4^)	1.02 × 10^−5^ (0, 7.90 × 10^−5^)	3.63 × 10^−4^ (0, 8.22 × 10^−4^)	1.93 × 10^−5^ (0, 4.19 × 10^−5^)	9.46 × 10^−4^ (7.76 × 10^−5^, 1.26 × 10^−3^)
	PFLX	2.74 × 10^−6^ (0, 1.06 × 10^−5^)	9.63 × 10^−4^ (0, 1.38 × 10^−4^)	3.42 × 10^−6^ (0, 2.14 × 10^−5^)	2.83 × 10^−6^ (0, 1.42 × 10^−5^)	2.73 × 10^−5^ (0, 1.07 × 10^−4^)	7.50 × 10^−5^ (0, 1.97 × 10^−4^)	3.78 × 10^−5^ (0, 1.24 × 10^−4^)	3.66 × 10^−6^ (0, 2.82 × 10^−5^)	4.33 × 10^−4^ (0, 1.03 × 10^−3^)	2.08 × 10^−6^ (0, 1.31 × 10^−5^)	1.45 × 10^−3^ (3.23 × 10^−5^, 4.32 × 10^−4^)
	NOR	2.74 × 10^−6^ (0, 1.06 × 10^−5^)	3.15 × 10^−5^ (0, 1.39 × 10^−4^)	3.42 × 10^−6^ (0, 2.14 × 10^−5^)	2.84 × 10^−6^ (0, 1.43 × 10^−5^)	2.73 × 10^−5^ (0, 1.08 × 10^−4^)	7.56 × 10^−5^ (0, 1.97 × 10^−4^)	3.78 × 10^−5^ (0, 1.24 × 10^−4^)	3.66 × 10^−6^ (0, 2.82 × 10^−5^)	4.02 × 10^−4^ (0, 8.84 × 10^−4^)	2.07 × 10^−6^ (0, 1.30 × 10^−5^)	2.17 × 10^−4^ (3.13 × 10^−5^, 4.24 × 10^−4^)
	LOM	1.53 × 10^−6^ (0, 5.93 × 10^−6^)	1.76 × 10^−5^ (0, 7.77 × 10^−5^)	1.92 × 10^−6^ (0, 1.20 × 10^−5^)	1.59 × 10^−6^ (0, 7.98 × 10^−6^)	1.53 × 10^−5^ (0, 5.91 × 10^−5^)	4.24 × 10^−5^ (0, 1.10 × 10^−4^)	2.12 × 10^−5^ (0, 6.95 × 10^−5^)	2.05 × 10^−6^ (0, 1.58 × 10^−5^)	3.60 × 10^−4^ (0, 8.23 × 10^−4^)	1.18 × 10^−6^ (0, 7.41 × 10^−6^)	1.19 × 10^−4^ (1.97 × 10^−5^, 2.32 × 10^−4^)

ENR: enrofloxacin; CIP: ciprofloxacin; OFL: ofloxacin; PFLX: pefloxacin; NOR: norfloxacin; LOM: lomefloxacin.

**Table A7:** HI of different QNs in various food categories (mean, 95%CI).

Age	Food Category										
	Beef	Chicken	Crustaceans	Duck	Eggs	Fish	Milk	Mollusk	Pork	Viscera	Total
3–6	4.38 × 10^−5^ (0, 1.85 × 10^−4^)	4.19 × 10^−3^ (0, 2.14 × 10^−3^)	4.09 × 10^−4^ (0, 4.97 × 10^−3^)	3.40 × 10^−4^ (0, 2.24 × 10^−4^)	4.86 × 10^−5^ (0, 1.99 × 10^−4^)	6.05 × 10^−3^ (0, 2.35 × 10^−2^)	1.74 × 10^−3^ (0, 4.97 × 10^−3^)	9.01 × 10^−4^ (0, 4.35 × 10^−4^)	5.04 × 10^−4^ (0, 1.36 × 10^−3^)	1.60 × 10^−3^ (0, 1.23 × 10^−3^)	1.94 × 10^−2^ (0, 6.56 × 10^−2^)
7–17	3.36 × 10^−5^ (0, 1.25 × 10^−4^)	3.71 × 10^−3^ (0, 1.58 × 10^−3^)	3.99 × 10^−4^ (0, 4.17 × 10^−3^)	1.97 × 10^−4^ (0, 1.97 × 10^−4^)	2.38 × 10^−5^ (0, 9.00 × 10^−5^)	4.77 × 10^−3^ (0, 1.59 × 10^−2^)	7.56 × 10^−4^ (0, 2.24 × 10^−3^)	9.79 × 10^−4^ (0, 4.17 × 10^−4^)	3.71 × 10^−4^ (0, 1.02 × 10^−3^)	1.82 × 10^−3^ (0, 1.05 × 10^−3^)	1.41 × 10^−2^ (0, 4.46 × 10^−2^)
18–59	2.60 × 10^−5^ (0, 9.82 × 10^−5^)	2.26 × 10^−3^ (0, 1.10 × 10^−3^)	3.31 × 10^−4^ (0, 3.63 × 10^−3^)	4.26 × 10^−4^ (0, 1.51 × 10^−4^)	1.46 × 10^−5^ (0, 5.65 × 10^−5^)	3.53 × 10^−3^ (0, 1.23 × 10^−2^)	3.06 × 10^−4^ (0, 1.05 × 10^−3^)	6.51 × 10^−4^ (0, 3.06 × 10^−4^)	2.65 × 10^−4^ (0, 7.07 × 10^−4^)	1.47 × 10^−3^ (0, 7.75 × 10^−4^)	1.05 × 10^−2^ (0, 3.70 × 10^−2^)
≥60	1.94 × 10^−5^ (0, 9.26 × 10^−5^)	1.53 × 10^−3^ (0, 8.59 × 10^−4^)	3.44 × 10^−4^ (0, 3.57 × 10^−3^)	4.75 × 10^−4^ (0, 1.28 × 10^−4^)	1.76 × 10^−5^ (0, 7.75 × 10^−5^)	4.24 × 10^−3^ (0, 1.42 × 10^−2^)	2.60 × 10^−4^ (0, 8.89 × 10^−4^)	8.33 × 10^−4^ (0, 2.91 × 10^−4^)	2.66 × 10^−4^ (0, 7.10 × 10^−4^)	1.32 × 10^−3^ (0, 9.25 × 10^−4^)	1.15 × 10^−2^ (0, 3.98 × 10^−2^)
Total	2.45 × 10^−5^ (0, 9.47 × 10^−5^)	1.92 × 10^−3^ (0, 1.10 × 10^−3^)	2.67 × 10^−4^ (0, 3.25 × 10^−3^)	2.82 × 10^−4^ (0, 1.44 × 10^−4^)	1.53 × 10^−5^ (0, 5.91 × 10^−5^)	3.34 × 10^−3^ (0, 1.23 × 10^−2^)	3.73 × 10^−4^ (0, 1.23 × 10^−3^)	9.98 × 10^−4^ (0, 3.00 × 10^−4^)	2.61 × 10^−4^ (0, 7.26 × 10^−4^)	1.35 × 10^−3^ (0, 7.92 × 10^−4^)	1.01 × 10^−2^ (0, 3.65 × 10^−2^)
